# Purification and Identification of Antioxidant Peptides from Protein Hydrolysate of Scalloped Hammerhead (*Sphyrna lewini*) Cartilage

**DOI:** 10.3390/md15030061

**Published:** 2017-03-01

**Authors:** Xue-Rong Li, Chang-Feng Chi, Li Li, Bin Wang

**Affiliations:** 1 School of Marine Science and Technology, Zhejiang Ocean University, 1st Haidanan Road, Changzhi Island, Lincheng, Zhoushan 316022, China; xuerongl0312@163.com (X.-R.L.); wenwenlili@163.com (L.L.); 2 School of Food and Pharmacy, Zhejiang Ocean University, 1st Haidanan Road, Changzhi Island, Lincheng, Zhoushan 316022, China

**Keywords:** scalloped hammerhead (*Sphyrna lewini*), cartilage, peptide, antioxidant activity

## Abstract

The aim of this study was to purify and identify peptides with antioxidant properties from protein hydrolysate of scalloped hammerhead (*Sphyrna lewini*) cartilage. Cartilaginous proteins of the scalloped hammerhead were extracted by guanidine hydrochloride, and three antioxidant peptides, named enzymolysis peptide of scalloped hammerhead cartilage A (SCPE-A), SCPE-B and SCPE-C, were subsequently isolated from the hydrolysate of the cartilaginous proteins using ultrafiltration and chromatography. The amino acid sequences of SCPE-A, SCPE-B and SCPE-C were identified as Gly-Pro-Glu (GPE), Gly-Ala-Arg-Gly-Pro-Gln (GARGPQ), and Gly-Phe-Thr-Gly-Pro-Pro-Gly-Phe-Asn-Gly (GFTGPPGFNG), with molecular weights of 301.30 Da, 584.64 Da and 950.03 Da, respectively. As per in vitro activity testing, SCPE-A, SCPE-B and SCPE-C exhibited strong scavenging activities on 2,2-diphenyl-1-picrylhydrazyl radicals (DPPH•) (half elimination ratio (EC_50_) 2.43, 2.66 and 1.99 mg/mL), hydroxyl radicals (HO•) (EC_50_ 0.28, 0.21 and 0.15 mg/mL), 2,2′-azino-bis-3-ethylbenzothiazoline-6-sulfonic acid radicals (ABTS^+^•) (EC_50_ 0.24, 0.18 and 0.29 mg/mL), and superoxide anion radicals ($O_{2}^{-}$•) (EC_50_ 0.10, 0.14 and 0.11 mg/mL). In addition, SCPE-A showed inhibition activity similar to butylated hydroxytoluene (BHT) in lipid peroxidation in a linoleic acid model system. The amino acid residues of Gly, Pro and Phe could positively influence the antioxidant activities of GPE, GARGPQ and GFTGPPGFNG. These results suggested that GPE, GARGPQ and GFTGPPGFNG might serve as potential antioxidants and be used as food additives and functional foods.

## 1. Introduction

Oxidation is an important factor in the food industry because it causes a loss of nutrition, color and functionality, as well as undesirable off-flavors and toxic compounds, which further induce the deterioration of food. Furthermore, the accumulation of toxic products is dangerous to the health of consumers [1,2]. Therefore, the inhibition of free radical formation and oxidation reactions play an important role in preventing or retarding the autoxidation of food components [3]. Many synthetic antioxidants, including butylated hydroxytoluene (BHT), butylated hydroxyanisole (BHA), and tertiary butylhydroquinone (TBHQ), are widely used in the food industry for preservation and to retard lipid oxidation [4,5]. However, the dosages of the synthetic antioxidants are under strict regulation due to their potential health hazards and toxic effects [6,7]. Therefore, there has been a large amount of interest in researching safe antioxidants from natural sources as an alternative to synthetic antioxidants [8].

In recent years, peptides with different activities, including anticancer, antioxidant, antimicrobial, antihypertensive, and mineral-binding properties, have been isolated from various bioresources, such as byproducts from the fish processing industry [9]. Antioxidant peptides have drawn great attention and have been extensively reported as free radical scavengers, peroxide decomposers, metal inactivators and oxygen inhibitors to protect food and organisms from reactive oxygen species (ROS) [8,10]. Arg-Gln-Ser-His-Phe-Ala-Asn-Ala-Gln-Pro (RQSHFANAQP), with molecular weight (MW) 1155 Da, from the protein hydrolysate of chickpeas showed significant dose-dependent scavenging activities on hydroxyl radicals (HO•) (EC_50_ 2.03 μmol/mL), 2,2-diphenyl-1-picrylhydrazyl radicals (DPPH•) (EC_50_ 3.15 μmol/mL) and 2, 2′-azino-bis-3-ethylbenzothiazoline-6-sulfonic acid radicals (ABTS^+^•) (EC_50_ 2.31 μmol/mL) [10]. Asp-Leu-Glu-Glu (DLEE), with MW 504.2 Da, was confirmed to be one of the main antioxidant peptides generated in dry-cured Xuanwei ham, and its DPPH• scavenging rate was 74.45% at 0.5 mg/mL [9]. Phe-Ile-Met-Gly-Pro-Tyr (FIMGPY), Gly-Pro-Ala-Gly-Asp-Tyr (GPAGDY) and Ile-Val-Ala-Gly-Pro-Gln (IVAGPQ), with MWs of 726.90 Da, 578.58 Da and 583.69 Da, respectively, showed strong scavenging activities on DPPH• (EC_50_ 3.5768, 6.0147 and 6.733 M), HO• (EC_50_ 4.1821, 6.7752 and 8.6176 M), superoxide anion radicals ($O_{2}^{-}$•) (EC_50_ 2.2149, 2.8691 and 3.1181 M) and ABTS^+^• (EC_50_ 1.4307, 1.3308 and 2.2101 M) [11]. In addition, FIMGPY induced HeLa cell apoptosis by up-regulating the Bax (B-cell lymphoma 2 (Bcl-2) assaciated X protein)/Bcl-2 ratio and caspase-3 activation [12]. Structure–activity studies on the antioxidant peptides suggested that the peptide length and the composition and position of amino acids in a peptide sequence are important determinants of the bioactivity of a specific peptide [8,13,14].

Cartilage is a form of connective tissue that is chemically abundant in bioactive components, and many cartilaginous proteins, low MW proteins, glycoproteins, and peptides have been prepared from various soft bone fish sources, such as bamboo and blacktip sharks [15], *Prionace glauca* [16], Amur sturgeon [17], spotless smoothhound [7], and silvertip shark [18]. Current research has shown that those active substances could be used as angiogenesis inhibitors, tumor cells inhibitory factors, antioxidants, immune regulatory factors, and anti-invasion factors for the treatment of some diseases, especially in tumor therapy and prevention. Scalloped hammerhead (*Sphyrna lewini*), belonging to the family Triakidae, is a commercially valuable fishery resource. At present, large quantities of muscle protein and cartilages (except the cartilages from fins) from scalloped hammerhead are not used efficiently. In our previous research, three antioxidant peptides, Trp-Asp-Arg (WDR), Pro-Tyr-Phe-Asn-Lys (PYFNK) and Leu-Asp-Lys (LDK), were isolated from the hydrolysate of scalloped hammerhead muscle, and WDR, PYFNK and LDK exhibited good scavenging activities on DPPH• (EC_50_ 3.63, 4.11 and 3.06 mg/mL), HO• (EC_50_ 0.15, 0.24 and 0.17 mg/mL), ABTS^+^• (EC_50_ 0.34, 0.12 and 0.19 mg/mL), and $O_{2}^{-}$• (EC_50_ 0.09, 0.11 and 0.12 mg/mL) [19,20]. Acid-soluble collagen and its hydrolysate were prepared from scalloped hammerhead cartilage [21]. However, to the best of our knowledge, there is no research focusing on the antioxidative peptides in scalloped hammerhead cartilage. Thus, in this study, three novel antioxidant peptides were isolated from the protein hydrolysate of scalloped hammerhead cartilage and their antioxidant activities were evaluated by DPPH•, HO•, $O_{2}^{-}$•, and ABTS^+^• scavenging and lipid peroxidation inhibition assays.

## 2. Results and Discussion

### 2.1. Preparation of the Protein Hydrolysate of Scalloped Hammerhead Cartilage (SHCH)

Proteins in raw and processed foods possess antioxidant peptide sequences and structural domains, and enzymatic hydrolysis is considered as an attractive way for releasing those active fragments without impairing their nutritional value and without leaving residual organic solvents and toxic chemicals in the final product [22]. In addition, protein hydrolysates using different proteases exhibit different antioxidant activities against various antioxidant systems because the peptides are different in terms of chain length and amino acid sequence [1,23]. Therefore, five proteases including papain, alcalase, trypsin, pepsin, and neutrase were used to hydrolyze the cartilaginous proteins of scalloped hammerhead. HO• scavenging assay is quick, convenient, and efficient in predicting the antioxidant activities of protein hydrolysates and purified peptides. As a consequence, HO• was used to evaluate the antioxidant activity of compounds to act as free radical scavengers or hydrogen donors, and the results are shown in Table 1.

The yield and degree of hydrolysis (DH%) of trypsin hydrolysate was 2.11 ± 0.11 g/100 g cartilage and 23.72% ± 0.31%, respectively, which was higher than for papain hydrolysate, alcalase hydrolysate, pepsin hydrolysate, and neutrase hydrolysate. The result indicated that trypsin could more effectively hydrolyze the proteins from scalloped hammerhead cartilages than the other four proteases. Furthermore, trypsin hydrolysate (SHCH) showed a significantly higher HO• scavenging activity (*p* < 0.05) with 62.38% ± 1.67% at 15 mg/mL, whereas papain hydrolysate showed a significantly lower HO• scavenging activity (*p* < 0.05) at 34.85% ± 1.05%. Based on these data, the protein hydrolysate of scalloped hammerhead cartilage produced by trypsin was named SHCH and was selected for follow-up studies.

### 2.2. Purification of the Antioxidant Peptides from SHCH

#### 2.2.1. Ultrafiltration

**Figure 1:**
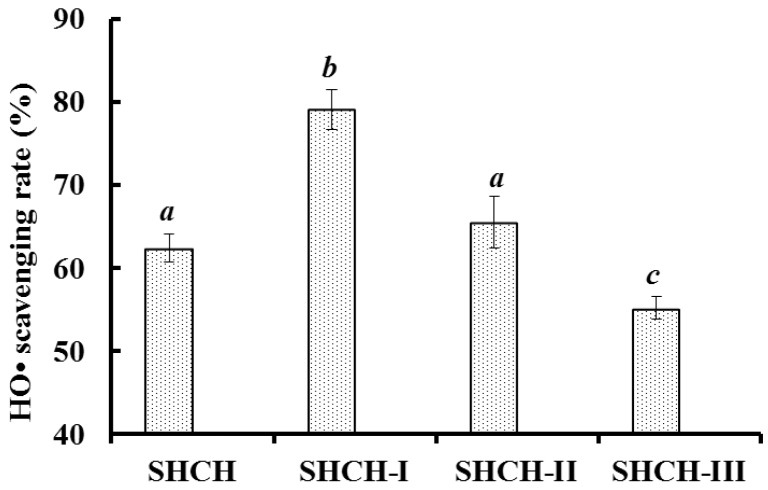
HO• scavenging activities of trypsin hydrolysate (SHCH) and its three fractions at 15 mg protein/mL. All data are presented as the mean ± standard deviation (SD) of triplicate results. ^*a*–*c*^ Values with same letters indicate no significant difference for each group of samples at the same concentration (*p* > 0.05).

Protein hydrolysate is a complex mixture of active and inactive peptides (of various sizes) and amino acid compositions, and ultrafiltration membrane technology is an important method for the fractionation of protein hydrolysate and the enrichment of peptides with specific MW ranges [1,5]. SHCH was fractionated by ultrafiltration using two molecular weight cut-off (MWCO) membranes (10 and 3 kDa), and three fractions, SHCH-I (MW < 3 kDa), SHCH-II (3 kDa < MW < 10 kDa), and SHCH-III (MW > 10 kDa), were prepared. As shown in Figure 1, the HO• scavenging activity of SHCH-I was 79.10% ± 2.38% at 15 mg protein/mL, which was significantly stronger than those of SHCH, SHCH-II, and SHCH-III (*p* < 0.05). The MW of peptides plays a critical role in bioactivity, and protein hydrolysates with smaller MW usually exhibited higher antioxidant activity than larger MW hydrolysates [4,5]. SHCH-I, which is abundant in smaller MW peptides, showed high HO• scavenging activity, and the result was in agreement with other reports that the ultrafiltration fractions of protein hydrolysates with lower MW could more effectively interact with the free radicals interfering in oxidative processes [6,9].

#### 2.2.2. Anion-Exchange Chromatography

**Figure 2:**
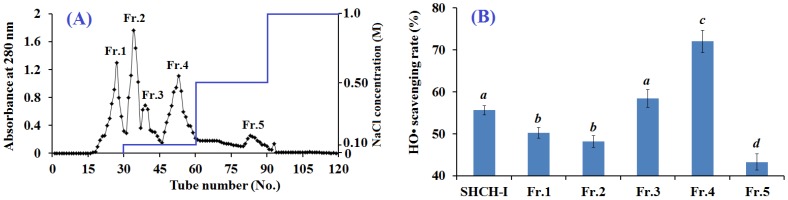
Elution profile of SHCH-I in DEAE-52 cellulose chromatography (**A**); and the HO• scavenging rate (%) of different fractions of SHCH-I at 10 mg protein/mL (**B**). All data are presented as the mean ± standard deviation (SD) of triplicate results. ^*a*–*d*^ Values with same letters indicate no significant difference for each group of samples at the same concentration (*p* > 0.05). Fr: separated fractions.

Ion-exchange chromatography is used to separate the charged molecules based on their affinity to the ion exchanger (anion and/or cation exchange resins), and their interaction was determined by the number and location of the charges on the molecules [5]. SHCH-I was loaded onto a Diethylaminoethyl cellulose 52 (DEAE-52) cellulose anion-exchange column and separated by stepwise elution using deionized water and 0.1, 0.5, and 1.0 M NaCl (Figure 2A). Five separated fractions (Fr.1 to Fr.5) were collected. Their HO• scavenging activities were measured and are shown in Figure 2B. The HO• scavenging rate of Fr.4 reached 72.03% ± 2.64% at 10 mg protein/mL, and it exhibited significantly more efficient antioxidant activity than the other fractions (*p* < 0.05). Peptides with basic and/or hydrophobic amino acid residues, such as His, Lys and Pro, are thought to have strong antioxidant activities [24]. Therefore, anion and cation exchange resins have been widely used to purify antioxidant peptides from protein hydrolysates [25,26,27]. The present data showed that Fr.4 had the strongest HO• scavenging activity and was selected for further purification.

#### 2.2.3. Gel Filtration Chromatography

**Figure 3:**
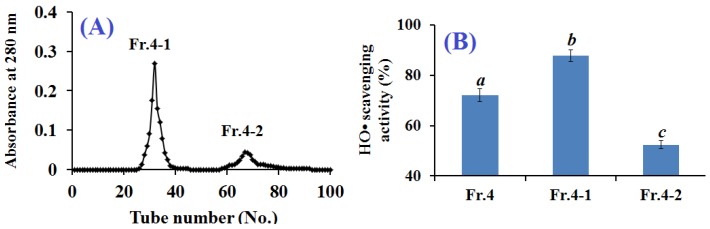
Elution profile of Fr.4 in Sephadex G-15 chromatography (**A**) and HO• scavenging activity of Fr.4 and its fractions at 5 mg protein/mL (**B**). All data are presented as the mean ± SD of triplicate results. ^*a*–*c*^ Values with same letters indicate no significant difference for each group of samples at the same concentration (*p* > 0.05).

Molecular size is an important determinant of the bioactivity of a specific peptide [8]. Therefore, gel filtration chromatography is an important method to purify peptides. Fr.4 was loaded onto a Sephadex G-15 column and separated into two fractions of Fr.4-1 and Fr.4-2 (Figure 3A). Each fraction was collected, lyophilized, and evaluated for HO• scavenging activity. As shown in Figure 3B, the HO• scavenging rate of Fr.4-1 reached 87.80% ± 2.24% at 5 mg protein/mL and was higher than those of Fr.4 (72.03% ± 2.64%) and Fr.4-2 (52.38% ± 1.62%). Therefore, Fr.4-1 was selected for further purification by RP-HPLC.

#### 2.2.4. Isolation of Peptides from Fr.4-1 by Reversed-Phase High Performance Liquid Chromatography (RP-HPLC)

**Figure 4:**
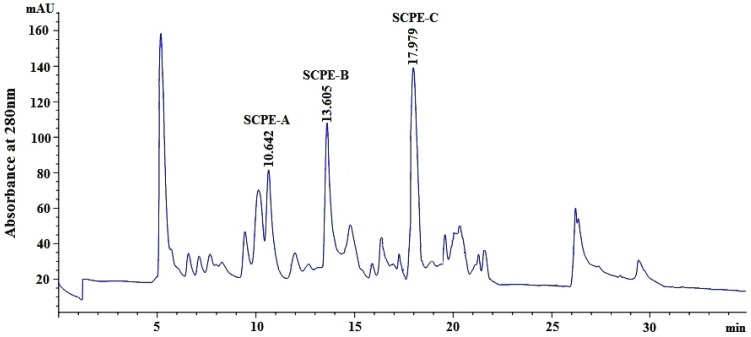
RP-HPLC profile of Fr.4-1 on a Zorbax C18 column with a linear gradient of acetonitrile (0%–50% for 32 min) containing 0.1% trifluoroacetic acid (TFA) at a flow rate of 0.8 mL/min.

The hydrophobic and hydrophilic properties of peptides play a key role in their retention time (RT) on an RP-HPLC column, and the RT can be adjusted by changing the ratio of polar (water) and nonpolar (methanol, acetonitrile) solvents [11]. Using an ultrafiltration membrane system, anion-exchange chromatography and gel filtration chromatography, Fr.4-1, which had the highest HO• scavenging activity among all fractions, was separated using RP-HPLC on a Zorbax C-18 column, and the eluted fractions were collected separately according to the chromatographic peaks (Figure 4). Three fractions, referred to as enzymolysis peptide of scalloped hammerhead cartilage A (SCPE-A), SCPE-B and SCPE-C, with RTs of 10.642, 13.605, and 17.979 min, respectively, showed high antioxidant activities, and their HO• scavenging rates reached 80.7% ± 1.22%, 75.4% ± 2.33%, and 92.2% ± 3.44%, respectively, at 3.0 mg/mL. Therefore, SCPE-A, SCPE-B and SCPE-C were collected for further research.

### 2.3. Molecular Mass and Amino Acid Sequences of the Purified Peptides

**Figure 5:**
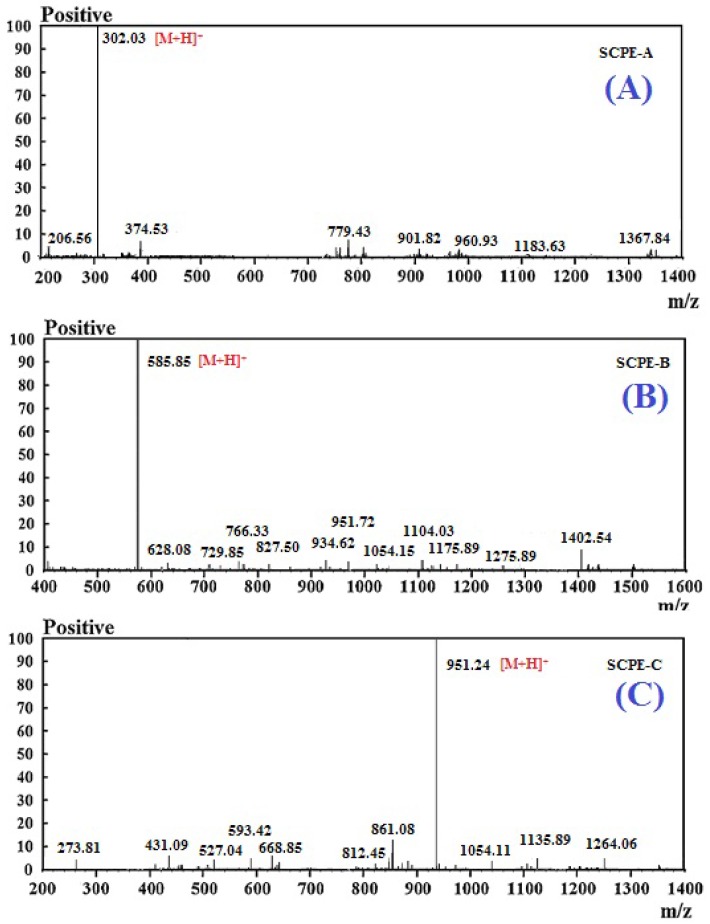
Mass spectrograms of SCPE-A, SCPE-B and SCPE-C.

The properties of peptides are related to their composition, structure, MW, amino acid sequence and hydrophobicity. Considering the radical-scavenging ability, the amino acid sequences and molecular mass of the three isolated peptides were analyzed using a protein sequencer and quadrupole-time of flight mass spectrometry (Q-TOF MS), respectively. The mass spectra of the three isolated peptides were shown in Figure 5. The amino acid sequences of SCPE-A, SCPE-B and SCPE-C were identified as Gly-Pro-Glu (GPE), Gly-Ala-Arg-Gly-Pro-Gln (GARGPQ), and Gly-Phe-Thr-Gly-Pro-Pro-Gly-Phe-Asn-Gly (GFTGPPGFNG), with molecular masses of 301.30 Da, 584.64 Da and 950.03 Da, respectively, which were in agreement with the theoretical masses of 301.27 Da, 584.64 Da, and 949.12 Da, respectively.

### 2.4. Antioxidant Activity of SCPE-A, SCPE-B and SCPE-C

#### 2.4.1. DPPH• Scavenging Activity

**Figure 6:**
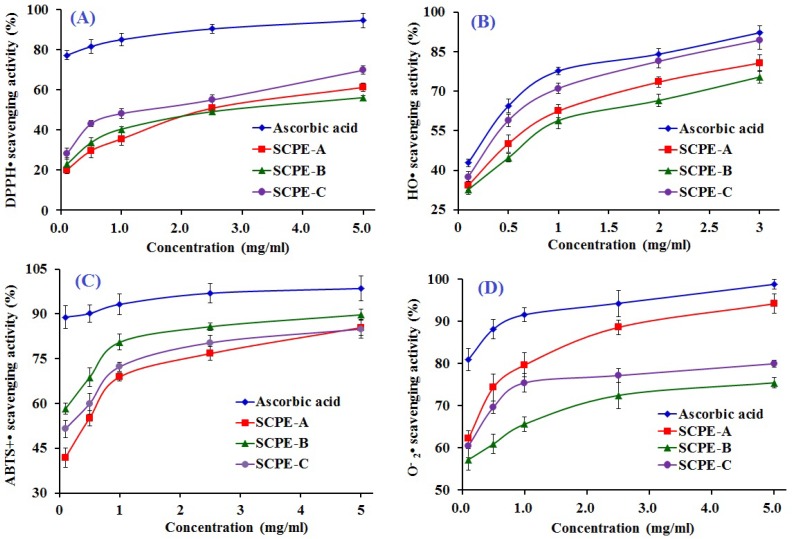
DPPH• (**A**); HO• (**B**); ABTS^+^• (**C**); and $O_{2}^{-}$• (**D**) scavenging activities of SCPE-A, SCPE-B and SCPE-C. All data are presented as the mean ± SD of triplicate results. DPPH•: 2,2-diphenyl-1-picrylhydrazyl radicals; ABTS^+^•: 2, 2′-azino-bis-3-ethylbenzothiazoline-6-sulfonic acid radicals; $O_{2}^{-}$•: superoxide anion radicals.

DPPH is a relatively stable organic radical that can be scavenged by accepting a proton-donating substance (H^+^), which reduces the absorbance at 517 nm because the solution color changes from deep purple to yellow [19]. As shown in Figure 6A, SCPE-A, SCPE-B and SCPE-C showed dose-dependent anti-DPPH• activity, with EC_50_ values of 2.43, 2.43, and 1.99 mg/mL, respectively, and SCPE-C exhibited the highest radical-scavenging activity among all samples, except the positive control of ascorbic acid. The EC_50_ of SCPE-C was lower than that of Pro-Ser-Tyr-Val (PSYV) (17.0 mg/mL) [28], Thr-Thr-Ala-Asn-Ile-Glu-Asp-Arg-Arg (TTANIEDRR) (2.503 mg/mL) [26], Phe-Leu-Asn-Glu-Phe-Leu-His-Val (FLNEFLHV) (4.950 mg/mL) [29], Trp-Glu-Gly-Pro-Lys (WEGPK) (4.438 mg/mL), Gly-Val-Pro-Leu-Thr (GVPLT) (4.541 mg/mL) [5], Gly-Phe-Gly-Pro-Leu (GFGPL) (2.249 mg/mL), Val-Gly-Gly-Arg-Pro (VGGRP) (2.937 mg/mL) [30], FIMGPY (2.60 mg/mL), GPAGDY (3.48 mg/mL), IVAGPQ (3.93 mg/mL) [11], WDR(3.63 mg/mL), PYFNK (4.11 mg/mL) and LDK (3.06 mg/mL) [19,20] from the protein hydrolysates of loach, blue mussel, salmon, bluefin leatherjacket, grass carp skin, skate (*Raja porosa*) cartilage and scalloped hammerhead muscle, but it was higher than that of Gly-Ser-Gln (GSQ) (0.61 mg/mL) [31], Pro-Ile-Ile-Val-Tyr-Trp-Lys (PIIVYWK) (0.713 mg/mL), Phe-Ser-Val-Val-Pro-Ser-Pro-Lys (FSVVPSPK) (0.937 mg/mL) [29], Pro-Tyr-Ser-Phe-Lys (PYSFK) (1.575 mg/mL) [30], His-Phe-Gly-Asp-Pro-Phe-His (HFGDPFH) (0.20 mg/mL) [32], Phe-Leu-Pro-Phe (FLPF) (0.789 mg/mL), Leu-Pro-Phe (LPF) (0.777 mg/mL) and Leu-Leu-Pro-Phe (LLPF) (1.084 mg/mL) [33] from the protein hydrolysates of Chinese leek, blue mussel, grass carp skin, mussel sauce and corn gluten meal. Therefore, the present results suggested that SCPE-A, SCPE-B and SCPE-C were DPPH• inhibitors and primary antioxidants that reacted with free radicals.

#### 2.4.2. HO• Scavenging Activity

HO• is highly reactive and consequently short-lived and can damage virtually all types of macromolecules, including carbohydrates, nucleic acids, lipids, and proteins [5]. The HO• scavenging activity of SCPE-A, SCPE-B and SCPE-C was dose-dependent at the test concentrations, as shown in Figure 6B. The EC_50_ values of SCPE-A, SCPE-B and SCPE-C were 0.28, 0.21, and 0.15 mg/mL, respectively, and SCPE-C exhibited the highest HO• scavenging activity. The EC_50_ of SCPE-C was lower than that of PYFNK (0.24 mg/mL), LDK (0.17 mg/mL) [19,20], Leu-Gly-Leu-Asn-Gly-Asp-Asp-Val-Asn (LGLNGDDVN) (0.687 mg/mL) [34], PSYV (2.64 mg/mL) [28], HFGDPFH (0.50 mg/mL) [32], Phe-Pro-Glu-Leu-Leu-Ile (FPELLI) (0.57 mg/mL) and Val-Phe-Ala-Ala-Leu (VFAAL) (0.31 mg/mL) [4], as well as that of Tyr-Pro-Pro-Ala-Lys (YPPAK) (0.228 mg/mL) [23], Pro-Ser-Lys-Tyr-Glu-Pro-Phe-Val (PSKYEPFV) (2.86 mg/mL) [35], PYSFK (2.283 mg/mL), GFGPL (1.612 mg/mL), VGGRP (2.055 mg/mL) [30], Tyr-Leu-Gly-Ala-Lys (YLGAK) (scavenging rate: 45.14% at 0.5 mg/mL), Gly-Gly-Leu-Glu-Pro-Ile-Asn-Phe-Gln (GGLEPINFQ) (scavenging rate: 41.07% at 0.5 mg/mL) [36], Asn-Gly-Leu-Glu-Gly-Leu-Lys (NGLEGLK) (0.313 mg/mL), Asn-Ala-Asp-Phe-Gly-Leu-Asn-Gly-Leu-Glu-Gly-Leu-Ala (NADFGLNGLEGLA) (0.612 mg/mL) [32], FIMGPY (3.04), GPAGDY (3.92 mg/mL) and IVAGPQ (5.03 mg/mL) [11] from the protein hydrolysates of scalloped hammerhead muscle, conger eel, weatherfish loach, mussel sauce, Chinese cherry seeds, blue mussel, grass carp, egg white, giant squid and skate (*R. porosa*) cartilage. The three isolated peptides, especially SCPE-C, revealed good HO• scavenging activity, which demonstrated that it could serve as a scavenger to reduce or eliminate the damage induced by HO• in foods and biological systems.

#### 2.4.3. ABTS^+^• Scavenging Activity

The ABTS^+^• scavenging assay is a sensitive method to determine the antioxidant capacity of bioactive compounds, in which sodium persulfate converts ABTS to its radical cation with a blue color and an absorption maximum of 734 nm, and the blue ABTS^+^• is converted back to its colorless neutral form when ABTS^+^• is reactive towards an antioxidant [10,37,38]. The abilities of SCPE-A, SCPE-B and SCPE-C to scavenge ABTS^+^• in comparison with ascorbic acid were investigated, and dose-related effects were observed at different peptide concentrations ranging from 0 to 5.0 mg/mL (Figure 6C). SCPE-B, with an EC_50_ of 0.18 mg/mL, showed the strongest scavenging activity on ABTS^+^• among the protein hydrolysate, fractions, and prepared peptides at all tested concentrations. The EC_50_ of SCPE-B was lower than those of WDR (0.34 mg/mL) [19], LDK (0.19 mg/mL) [20], FLNEFLHV (1.548 mg/mL) [29], FPELLI (0.40 mg/mL) and VFAAL (0.38 mg/mL) [4], FLPF (1.497 mg/mL), LPF (1.013 mg/mL), LLPF (1.031 mg/mL) [33], GFGPL (0.328 mg/mL), VGGRP (0.465 mg/mL) [30], WEGPK (5.407 mg/mL), Gly-Pro-Pro (GPP) (2.472 mg/mL), GVPLT (3.124 mg/mL) [6], FIMGPY (1.04 mg/mL), GPAGDY (0.77 mg/mL) and IVAGPQ (1.29 mg/mL) [11] from the protein hydrolysates of scalloped hammerhead muscle, salmon, Chinese cherry seeds, corn gluten meal, grass carp skin, bluefin leatherjacket heads and skate cartilage. The present results indicated that SCPE-A, SCPE-B and SCPE-C could strongly donate electrons or hydrogen atoms to inactivate ABTS^+^•.

#### 2.4.4. $O_{2}^{-}$• Scavenging Activity

$O_{2}^{-}$• is the most common free radical and can produce hydrogen peroxide and hydroxyl radicals through dismutation and other reactions in vivo, which can cause damage to DNA, proteins and cell membranes. The $O_{2}^{-}$• scavenging activities of SCPE-A, SCPE-B and SCPE-C were studied, and the dose–effect relations were observed as the concentration gradually increased from 0.1 to 5.0 mg/mL (Figure 6D). The EC_50_ values of SCPE-A, SCPE-B and SCPE-C were 0.08, 0.14, and 0.11 mg/mL, respectively. SCPE-A showed stronger $O_{2}^{-}$• scavenging activity than SCPE-B and SCPE-C and reached 91.7% ± 2.58% scavenging activity at 5.0 mg/mL. The EC_50_ of SCPE-A was lower than that of WDR (0.09 mg/mL), PYFNK (0.11 mg/mL), LDK(0.12 mg/mL) [19,20], HFGDPFH (0.20 mg/mL) [32], GSQ (0.70 mg/mL) [31], Ser-Leu-Pro-Ile-Gly-Leu-Met-Ile-Ala-Met (SLPIGLMIAM) (0.3168 mg/mL) [39], YLGAK (scavenging rate: 36.27% at 1.0 mg/mL), GGLEPINFQ (scavenging rate: 32.05% at 1.0 mg/mL) [36], His-Asp-His-Pro-Val-Cys (HDHPVC) (0.265 mg/mL) and His-Glu-Lys-Val-Cys (HEKVC) (0.235 mg/mL) [40], Tyr-Leu-Met-Arg (YLMR) (0.450 mg/mL), Val-Leu-Tyr-Glu-Glu (VLYEE) (0.693 mg/mL), Met-Ile-Leu-Met-Arg (MILMR) (0.993 mg/mL) [41], FIMGPY (1.61 mg/mL), GPAGDY (1.66 mg/mL) and IVAGPQ (1.82 mg/mL) [11] from the protein hydrolysates of scalloped hammerhead muscle, mussel sauce, Chinese leek seeds, *Mytilus coruscus*, egg white, round scad, croceine croaker muscle and skate cartilage. $O_{2}^{-}$• could be catalyzed into hydrogen peroxide and oxygen by superoxide dismutase (SOD) with a reaction rate 10,000-fold higher than that of spontaneous dismutation in an organism [19]. Therefore, SCPE-A, SCPE-B and SCPE-C might have high antioxidant activity similar to SOD and could be applied as $O_{2}^{-}$• scavengers in biological systems.

#### 2.4.5. Lipid Peroxidation Inhibition Assay

**Figure 7:**
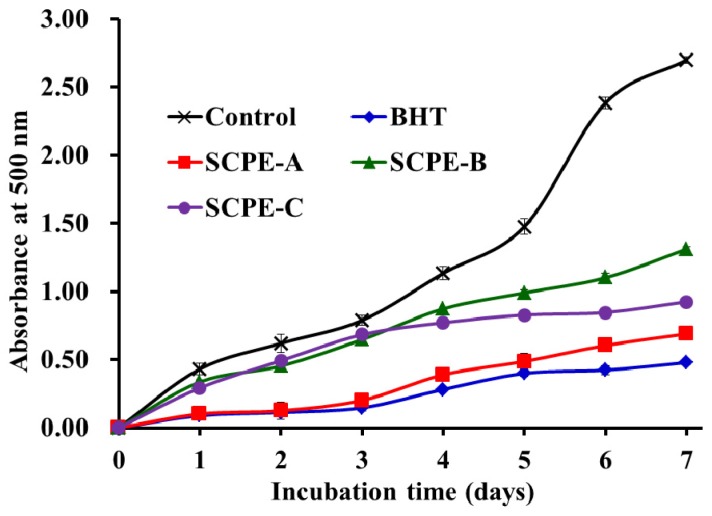
Lipid peroxidation inhibition assays of SCPE-A, SCPE-B and SCPE-C. All data are presented as the mean ± SD of triplicate results.

Scavenging activities on DPPH•, ABTS^+^•, HO• and $O_{2}^{-}$• have been widely used to assess the antioxidant capacities of protein hydrolysates and peptides. However, each of these assays only measures an antioxidant property representing a different mechanism, which does not reflect the multiple mechanisms by which samples may act as antioxidants to retard or inhibit lipid oxidation in a food system [42]. Therefore, in this section, the ability of the soluble samples to suppress lipid peroxidation in a linoleic acid model system was investigated. Lipid peroxidation is a complex process that involves the formation and propagation of lipid radicals and lipid hydroperoxides, which are formed as the primary oxidation products in the presence of oxygen [43]. The inhibitory activities of SCPE-A, SCPE-B and SCPE-C were measured by lipid peroxidation in an in vitro model and were compared with the commercially available antioxidant BHT for 7 days. Figure 7 shows that SCPE-A had a similar inhibitory effect on lipid peroxidation as BHT and significantly retarded the lipid peroxidation compared with the control (without sample), SCPE-B and SCPE-C. In previous research, SCPE-A showed excellent scavenging activity on DPPH•, HO•, ABTS^+^• and $O_{2}^{-}$•, with EC_50_ values of 2.43, 0.28, 0.24, and 0.08 mg/mL, respectively. Therefore, the inhibition effect of lipid peroxidation caused by SCPE-A could be attributed to its radical-scavenging activity. In addition, SCPE-A may have potential applications in the food industry for retarding the production of unwanted off-flavors and toxic products.

### 2.5. Structure-Antioxidant Activity Relationship of Peptides

The structural characteristics of peptides provide guides for the evaluation of food-derived proteins as potential precursors of antioxidant peptides and predict the possible release of bioactive peptides from various proteins using an appropriate protease [1].

Many researchers found that the antioxidant activity of peptides was highly dependent on their amino acid sequence and composition. Chen et al. (2012) reported that the Gly residue may contribute significantly to antioxidant activity since the single hydrogen atom in the side chain of Gly serves as a proton-donating source and neutralizes active free radical species [44]. In addition, Nimalaratne et al. (2015) reported that the single hydrogen atom of Gly (G) can provide high flexibility to the peptide backbone and positively influence the antioxidant properties [8]. Therefore, Gly residues might be important contributors to the antioxidant activity of SCPE-A, SCPE-B, and SCPE-C because there are one, two and four Gly residues in their amino acid sequences, respectively.

The pyrrolidine ring of proline (P) can interact with the secondary structure of the peptide, thereby increasing the flexibility, and it is also capable of quenching singlet oxygen due to its low ionization potential [6]. Samaranayaka and Li-Chan (2011) reported that the Pro residue plays an important role in the antioxidant activity of the peptide purified from *Saccharomyces cerevisiae* protein hydrolysate [45]. Therefore, the one, one and two Pro residues in the amino acid sequences of SCPE-A, SCPE-B, and SCPE-C should enhance the radical-scavenging activities of the three peptides.

Aromatic amino acids, such as Phe, Tyr, His, and Trp, and hydrophobic amino acids, including Ala, Val, and Leu, have been reported to be critical to the antioxidant activities of peptides [1]. Huang et al. (2005) reported that amino acids with aromatic residues, such as Phe, Tyr and Trp, can quench free radicals by direct electron transfer [46]. The results from Guo et al. (2015) indicated that hydrophobic amino acids (e.g., Val, Ala, Leu) and aromatic amino acids (Phe, His, Tyr and Trp) can enhance the radical-scavenging abilities of peptides from Chinese cherry seeds [4]. Therefore, the presence of the one Ala residue and two Phe residues in the sequences of SCPE-B and SCPE-C, respectively, should have a positive impact on their radical-scavenging and lipid peroxidation inhibitory activities.

The presence of acidic and basic amino acids plays a critical role in the metal ion chelating activity, which is related to the carboxyl and amino groups in their side chains [47]. Similar results were reported by Memarpoor-Yazdi et al. (2012), who found that the basic (Arg) and acidic (Asp and Glu) amino acid residues in the sequences of Asn-Thr-Asp-Gly-Ser-Thr-Asp-Tyr-Gly-Ile-Leu-Gln-Ile-Asn-Ser-Arg (NTDGSTDYGILQINSR) and Leu-Asp-Glu-Pro-Asp-Pro-Leu-Ile (LDEPDPLI) were critical to their antioxidant activities [48]. Díaz, et al. (2003) found that Glu is an effective cation chelator that forms complexes with calcium, iron and zinc and may contribute to the antioxidant activity [49]. Therefore, Glu in SCPE-A and Arg in SCPE-B might be favorable to their antioxidant activities.

In addition, the antioxidant activities of peptides are dependent on their molecular size, and shorter peptides, especially peptides with 2–10 amino acid residues, have stronger radical-scavenging and lipid peroxidation inhibition activities than their parent native proteins or long-chain peptides [1,46]. SCPE-A, SCPE-B, and SCPE-C exhibited good antioxidant activities in the radical scavenging and lipid peroxidation inhibition assays, which suggested that the short SCPE-A, SCPE-B, and SCPE-C could interact more effectively and easily with free radicals and inhibit the propagation cycles of lipid peroxidation in the radical scavenging and lipid peroxidation model system [50]. However, SCPE-A had the strongest $O_{2}^{-}$• scavenging and lipid peroxidation inhibition activities, SCPE-B had the strongest scavenging activity on ABTS^+^•, and SCPE-C exhibited the highest DPPH• and HO• scavenging activities among all samples and fractions. The results indicated no consistent trends in the antioxidant capacities of SCPE-A, SCPE-B, and SCPE-C in different antioxidant assays. Therefore, more detailed study should be performed to clarify the relationship between the activity and structure of the three isolated peptides.

## 3. Experimental Section

### 3.1. Chemicals and Reagents

Scalloped hammerhead (*S. lewini*) was purchased from Fengmao market in Zhoushan City, Zhejiang Province, China. DEAE-52 cellulose and Sephadex G-15 were purchased from Shanghai Source Poly Biological Technology Co., Ltd. (Shanghai, China). Acetonitrile was of liquid chromatography (LC) grade and was purchased from Thermo Fisher Scientific Co., Ltd. (Shanghai, China). All other reagents used in the experiment were of analytical grade and were purchased from Sinopharm Chemical Reagent Co., Ltd. (Shanghai, China).

### 3.2. Preparation of the Protein Hydrolysate from Scalloped Hammerhead Cartilage

Frozen scalloped hammerhead cartilage was thawed, minced to homogenate and soaked in 1.0 M guanidine hydrochloride with a solid-to-solvent ratio of 1:5 (*w*/*v*) for 48 h with continuous stirring, and the liquid supernatant was collected by centrifugation at 12,000× *g* at 4 °C for 10 min. The resulting supernatant was dialyzed (MW 5 kDa) against 25 volumes of distilled water for 12 h, with the solution changed every 4 h, and the resulting dialysate was freeze-dried.

The freeze-dried sample was dissolved (5% *w*/*v*) in 0.2 M phosphate buffer solution (PBS, pH 7.2) and hydrolyzed for 4 h using neutrase at pH 7.0, 60 °C; alcalase at pH 8.0, 50 °C; trypsin at pH 8.0, 40 °C; pepsin at pH 2.0, 37 °C; or papain at pH 6.0, 50 °C, with a total enzyme dose of 2.5%. Enzymatic hydrolysis was stopped by heating for 10 min in boiling water, and the hydrolysate was centrifuged at 9000× *g* for 15 min. The supernatant was freeze-dried and stored at −20 °C for further analysis. The protein hydrolysate of scalloped hammerhead cartilage using trypsin was named SHCH.

### 3.3. Isolation of Peptides from SHCH

#### 3.3.1. Fractionation of SHCH by Ultrafiltration

SHCH was fractionated using ultrafiltration (8400, Millipore, Hangzhou, China) with 10 kDa and 3 kDa MW cutoff (MWCO) membranes (Millipore, Hangzhou, China). Three peptide fractions, named SHCH-I (MW < 3 kDa), SHCH-II (3 kDa < MW < 10 kDa) and SHCH-III (MW > 10 kDa), were collected and lyophilized.

#### 3.3.2. Anion-Exchange Chromatography

SHCH-I (5 mL, 40.0 mg/mL) was injected onto a DEAE-52 cellulose column (1.6 × 70 cm) that was pre-equilibrated with deionized water and stepwise eluted with 150 mL distilled water and 0.1, 0.5, and 1.0 M NaCl solution at a flow rate of 1.0 mL/min. Each eluted fraction (5 mL) was collected and measured at 280 nm, and five fractions (Fr.1–5) were pooled and lyophilized.

#### 3.3.3. Gel filtration Chromatography

Fr.4 (5 mL, 10.0 mg/mL) was fractionated on a Sephadex G-15 column (2.6 × 160 cm) eluted with deionized water at a flow rate of 0.6 mL/min. Each eluate (3 mL) was collected and monitored at 280 nm, and two fractions (Fr.4-1 and Fr.4-2) were lyophilized.

#### 3.3.4. RP-HPLC

Fr.4-2 was separated by RP-HPLC (Agilent 1260 HPLC, Agilent Ltd., Santa Rosa, CA, USA) on a Thermo C-18 column (4.6 × 250 mm, 5 μm) (Thermo Co., Ltd., Yokohama, Japan) using a linear gradient of acetonitrile (0%–50% in 0–32 min) in 0.1% trifluoroacetic acid at a flow rate of 0.8 mL/min. The eluate was analyzed at 280 nm, and three peptides (SCPE-A, SCPE-B and SCPE-C) were isolated and lyophilized.

### 3.4. Determination of the Amino Acid Sequence and Molecular Mass

The amino acid sequences of SCPE-A, SCPE-B and SCPE-C were determined on an Applied Biosystems 494 protein sequencer (Perkin Elmer/Applied Biosystems Inc., Foster City, CA, USA). The molecular masses were determined using a Q-TOF mass spectrometer coupled to an electrospray ionization source (ESI) (Micromass, Waters, Los Angeles, CA, USA).

### 3.5. Degree of Hydrolysis (DH)

DH analysis was performed according to the previously described method [49]. The hydrolysate (50 μL) was mixed with 0.5 mL of 0.2 M phosphate buffer, pH 8.2 and 0.5 mL of 0.05% trinitrobenzenesulfonic acid (TNBS) reagent. TNBS was freshly prepared before use by diluting with DI water. The mixture was incubated at 50 °C for 1 h in a water bath. The reaction was stopped by adding 1 mL of 0.1 M HCl and incubating at room temperature for 30 min. The absorbance was monitored at 420 nm. l-leucine was used as a standard. To determine the total amino acid content, mungbean meal was completely hydrolyzed with 6 M HCl with a sample to acid ratio of 1:100 at 120 °C for 24 h. DH (%) was calculated using the following equation:

DH = [(A_t_ − A_0_)/(A_max_ − A_0_)] × 100

where A_t_ was the amount of a-amino acids released at time t, A_0_ was the amount of a-amino acids in the supernatant at 0 h, and A_max_ was the total amount of a-amino acids obtained after acid hydrolysis at 120 °C for 24 h.

### 3.6. Antioxidant Activity

The radical (DPPH•, HO•, $O_{2}^{-}$•, and ABTS^+^•) scavenging activity and lipid peroxidation inhibition assays were performed according to previously reported methods [19,51], and the half elimination ratio (EC_50_) was defined as the concentration of a sample that caused a 50% decrease in the initial concentration of DPPH•, $O_{2}^{-}$•, HO•, and ABTS^+^•. The EC_50_ was calculated based on the linear relationship of the radical-scavenging rate and concentration of the samples.

#### 3.6.1. HO• Scavenging Activity

In this system, hydroxyl radicals are generated by the Fenton reaction. Hydroxyl radicals can oxidize Fe^2+^ into Fe^3+^, and only Fe^2+^ can combine with 1,10-phenanthroline to form a red compound (1,10-phenanthroline-Fe^2+^) with the maximum absorbance at 536 nm. The concentration of hydroxyl radical is reflected by the degree of decolorization of the reaction solution. Briefly, 1,10-phenanthroline solution (1.0 mL, 1.865 mM) and the sample (2.0 mL) were added into a screw-capped tube and mixed. The FeSO_4_·7H_2_O solution (1.0 mL, 1.865 mM) was then pipetted into the mixture. The reaction was initiated by adding 1.0 mL H_2_O_2_ (0.03% *v*/*v*). After being incubated at 37 °C for 60 min in a water bath, the absorbance of the reaction mixture was measured at 536 nm against a reagent blank. The reaction mixture without any antioxidant was used as the negative control, and mixture without H_2_O_2_ was used as the blank. The HO• scavenging activity was calculated by the following formula:

HO• scavenging activity (%) = [(A_s_ − A_n_)/(A_b_ − A_n_)] × 100%

where A_s_, A_n_, and A_b_ were the absorbance values determined at 536 nm of the sample, the negative control, and the blank after reaction, respectively.

#### 3.6.2. DPPH• Scavenging Activity

Two milliliters of deionized water containing different concentrations of samples were placed in cuvettes, and then 500 μL of ethanol solution of DPPH (0.02%) and 1.0 mL of ethanol were added into. A control sample containing DPPH solution without sample was also prepared. For the blank absorbance, DPPH solution was substituted with ethanol. The antioxidant activity of the sample was evaluated by the inhibition percentage of DPPH• with the following equation:

DPPH• scavenging activity (%) = (A_0_ + A′ − A)/A_0_ × 100%

where A was sample absorbance rate; A_0_ was the absorbance of control group; A′ was the blank absorbance.

#### 3.6.3. $O_{2}^{-}$• Scavenging Activity

In the experiment, superoxide anions were generated in 1 mL of nitrotetrazolium blue chloride (NBT) (2.52 mM), 1 mL of nicotinamide adenine dinucleotide (NADH) (624 mM) and 1 mL of different concentrations of samples. The reaction was initiated by adding 1 mL of phenazine methosulfate (PMS) solution (120 μg) to the reaction mixture. The absorbance was measured at 560 nm against the corresponding blank after 5 min of incubation at 25 °C. The capacity of scavenging the $O_{2}^{-}$• was calculated using the following equation:

$$O_{2}^{-}•\text{ scavenging activity }(\%)=[(A_{\mathrm{control}}-A_{\mathrm{sample}})/A_{\mathrm{control}}]\times 100\%$$

where A_control_ was the absorbance without sample and A_sample_ was the absorbance with sample.

#### 3.6.4. ABTS^+^• Scavenging Activity

The ABTS radical cation was generated by mixing ABTS stock solution (7 mM) with potassium persulphate (2.45 mM). The mixture was left in the dark at room temperature for 16 h. The ABTS radical solution was diluted in 5 mM phosphate buffered saline (PBS) pH 7.4, to an absorbance of 0.70 ± 0.02 at 734 nm. One milliliter of diluted ABTS radical solution was mixed with one milliliter of different concentrations of samples. Ten minutes later, the absorbance was measured at 734 nm against the corresponding blank. The ABTS^+^• scavenging activity of samples was calculated using the following equation:

ABTS^+^• scavenging activity (%) = [(A_control_ − A_sample_)/A_control_] × 100%

where A_control_ was the absorbance without sample and A_sample_ was the absorbance with sample.

#### 3.6.5. Lipid Peroxidation Inhibition Assay

A sample (5.0 mg) was dissolved in 10 mL of 50 mM phosphate buffer (pH 7.0), and added to a solution of 0.13 mL of linoleic acid and 10 mL of 99.5% ethanol. Then, the total volume was adjusted to 25 mL with deionized water. The mixture was incubated in a conical flask with a screw cap at 40 ± 1 °C in a dark room and the degree of oxidation was evaluated by measuring the ferric thiocyanate values. The reaction solution (100 μL) incubated in the linoleic acid model system was mixed with 4.7 mL of 75% ethanol, 0.1 mL of 30% ammonium thiocyanate, and 0.1 mL of 20 mM ferrous chloride solution in 3.5% HCl. After 3 min, the thiocyanate value was measured by reading the absorbance at 500 nm following color development with FeCl_2_ and thiocyanate at different intervals during the incubation period at 40 ± 1 °C.

### 3.7. Statistical Analysis

All experiments were performed in triplicate (*n* = 3), and the data are expressed as the mean ± standard deviation (SD). ANOVA was applied to analyze the data using SPSS 19.0 (SPSS Corporation, Chicago, IL, USA). Duncan’s multiple range test was used to measure the differences between the parameter means. The differences were considered significant if *p* < 0.05.

## 4. Conclusions

In this study, three new antioxidant peptides (SCPE-A, SCPE-B and SCPE-C) were isolated from the protein hydrolysate of scalloped hammerhead (*S. lewini*) cartilage by ultrafiltration and chromatography, and their amino acid sequences were identified as Gly-Pro-Glu (GPE), Gly-Ala-Arg-Gly-Pro-Gln (GARGPQ), and Gly-Phe-Thr-Gly-Pro-Pro-Gly-Phe-Asn-Gly (GFTGPPGFNG). SCPE-A, SCPE-B and SCPE-C exhibited strong radical scavenging and lipid peroxidation inhibition activities. These results suggested that the purified peptides from the protein hydrolysate of scalloped hammerhead cartilage may be applied as ingredients in functional foods in bioactive food products. Our subsequent studies will focus on the molecular mechanisms and the relationship between the antioxidant activity and structure of the three isolated peptides.

## Figures and Tables

**Table 1:** Hydroxyl radical (HO•) scavenging activity of the protein hydrolysate of scalloped hammerhead cartilage using different proteases (*c* = 15 mg protein/mL).

Protease	Enzymolysis Condition	Yields (g/100 g Cartilage)	Degree of Hydrolysis (DH%)	HO• Scavenging Rate (%)
Papain	pH 7.0, 60 °C, 4 h, total enzyme dose 2.5%	1.93 ± 0.08 ^a^	18.33 ± 0.25 ^a^	34.85 ± 1.05 ^a^
Alcalase	pH 8.0, 50 °C, 4 h, total enzyme dose 2.5%	1.96 ± 0.10 ^a,b^	21.37 ± 0.35 ^b,c^	54.76 ± 1.94 ^b^
Trypsin	pH 8.0, 40 °C, 4 h, total enzyme dose 2.5%	2.11 ± 0.11 ^b^	23.72 ± 0.31 ^c^	62.38 ± 1.67 ^c^
Pepsin	pH 2.0, 37 °C, 4 h, total enzyme dose 2.5%	1.99 ± 0.07 ^a,b^	21.58 ± 0.26 ^c^	55.47 ± 2.02 ^b^
Neutrase	pH 6.0, 50 °C, 4 h, total enzyme dose 2.5%	1.85 ± 0.06 ^a^	20.87 ± 0.36 ^b^	50.67 ± 1.85 ^d^

^a–d^ Values with different letters indicate significant differences at the same concentration (*p* < 0.05).
